# Enhanced Tumor Imaging Using Glucosamine-Conjugated Polyacrylic Acid-Coated Ultrasmall Gadolinium Oxide Nanoparticles in Magnetic Resonance Imaging

**DOI:** 10.3390/ijms23031792

**Published:** 2022-02-04

**Authors:** Shuwen Liu, Huan Yue, Son Long Ho, Soyeon Kim, Ji Ae Park, Tirusew Tegafaw, Mohammad Yaseen Ahmad, Seungho Kim, Abdullah Khamis Ali Al Saidi, Dejun Zhao, Ying Liu, Sung-Wook Nam, Kwon Seok Chae, Yongmin Chang, Gang Ho Lee

**Affiliations:** 1 Department of Chemistry, College of Natural Sciences, Kyungpook National University, Taegu 41566, Korea; liushuwen0701@gmail.com (S.L.); yuehuan888@gmail.com (H.Y.); sonlongh@gmail.com (S.L.H.); tirukorea@gmail.com (T.T.); yaseen.knu@gmail.com (M.Y.A.); abdullah_al_saidi@hotmail.com (A.K.A.A.S.); djzhao.chem@gmail.com (D.Z.); ly1124161@gmail.com (Y.L.); 2 Division of RI-Convergence Research, Korea Institute of Radiological and Medical Sciences (KIRAMS), Seoul 01817, Korea; ksy0188@kirams.re.kr (S.K.); jpark@kirams.re.kr (J.A.P.); 3 Department of Molecular Medicine, School of Medicine, Kyungpook National University, Taegu 41944, Korea; seungho5335@gmail.com (S.K.); nams@knu.ac.kr (S.-W.N.); 4 Department of Biology Education, Teachers’ College, Kyungpook National University, Taegu 41566, Korea; kschae@knu.ac.kr

**Keywords:** glucosamine, enhanced tumor imaging, GlcN effects, ultrasmall gadolinium oxide nanoparticle, magnetic resonance imaging

## Abstract

Owing to a higher demand for glucosamine (GlcN) in metabolic processes in tumor cells than in normal cells (i.e., GlcN effects), tumor imaging in magnetic resonance imaging (MRI) can be highly improved using GlcN-conjugated MRI contrast agents. Here, GlcN was conjugated with polyacrylic acid (PAA)-coated ultrasmall gadolinium oxide nanoparticles (UGONs) (d_avg_ = 1.76 nm). Higher positive (brighter or T_1_) contrast enhancements at various organs including tumor site were observed in human brain glioma (U87MG) tumor-bearing mice after the intravenous injection of GlcN-PAA-UGONs into their tail veins, compared with those obtained with PAA-UGONs as control, which were rapidly excreted through the bladder. Importantly, the contrast enhancements of the GlcN-PAA-UGONs with respect to those of the PAA-UGONs were the highest in the tumor site owing to GlcN effects. These results demonstrated that GlcN-PAA-UGONs can serve as excellent T_1_ MRI contrast agents in tumor imaging via GlcN effects.

## 1. Introduction

Tumor diagnosis is a major challenge in the medical field [1,2]. Among various imaging techniques, magnetic resonance imaging (MRI), a noninvasive imaging technique in which radiofrequency proton spin signals are processed, has been widely applied in tumor diagnosis because of its high anatomical resolution and sensitivity due to the ample existence of protons in the body [3,4].

Tumor imaging using MRI can be improved via active and/or passive tumor-targeting methods, such as drug delivery [5,6]. For active targeting [5,6,7], tumor-targeting ligands are conjugated with imaging agents, while for passive targeting [5,6,8], the enhanced permeability and retention (EPR) effects of nanoparticle imaging agents are utilized, implying that nanoparticle agents can allow active and passive targeting. In addition, tumor imaging can be improved using conjugation ligands, which are highly consumed in the metabolic process of tumor cells. For example, glucose as the food of cells is more highly consumed by tumor cells than by normal cells because of the higher metabolic activity of the tumor cells [9,10,11]. Glucosamine (GlcN), an analog of glucose (one –OH group in glucose is replaced with an –NH_2_ group in GlcN), is another example. It is found in glycosylated proteins and lipids in humans and animals [12]; it is consumed in GlcN metabolic processes in cells [13,14,15]. Glucose and GlcN are delivered to cells via various glucose transporters (GLUTs) and sodium glucose transporters (SGLTs) expressed on cell membranes [9,16], many of which are overexpressed on tumor cells to facilitate glucose and GlcN delivery [17]. Owing to the aforementioned higher GlcN demand by tumor cells than by normal cells (GlcN effects) and the –NH_2_ group of GlcN, which allows easy conjugation with the –COOH group of functional agents via an amide bond (–OH group of glucose is not easy for conjugation), GlcN has been widely applied as a conjugation ligand to increase delivery amounts of antitumor drugs [18,19,20,21], tumor imaging agents [21,22,23], and tumor therapeutic agents [24]. GlcN had been conjugated with various materials for drug delivery to tumor, tumor imaging, and tumor therapy. These included poly(amidoamine) dendrimers to deliver camptothecin antitumor drug to human lung tumor (A549) cells in vitro and in vivo [18], graphene quantum dots to deliver curcumin antitumor drug to human breast tumor (MCF-7) cells in vitro [19], niosomal formulation to deliver doxorubicin antitumor drug to skin melanoma tumor (B6F10) cells in vitro [20], InP/ZnS quantum dots to deliver doxorubicin to human lung epithelial tumor (A549) cells and human ovarian tumor (OVCAR-3) cells in vitro and to image tumor cells [21], near infrared fluorescent probes for breast [22] and prostate [23] tumor imaging in vitro and in vivo, and multifunctional doxorubicin loaded gadolinium/cobalt@iron oxide-dendrimer-nanoseeds for chemo-magneto hyperthermia treatment of human prostate tumor (PC3) cells in vitro [24].

Recently, GlcN has been applied as a conjugation ligand in MRI to improve imaging contrasts [25,26,27,28]. It was conjugated with micelles (<20 nm) to improve normal mice liver imaging [25], polycyclodextrin-coated large Gd_2_O_3_ nanoparticles (>10 nm) [26], Gd^3+^-based nanoporous silica nanoparticles (~100 nm) [27], and Gd-chelates [28] to improve mice tumor imaging. However, GlcN has not been tried as a conjugation ligand of ultrasmall gadolinium oxide nanoparticles (UGONs). Briefly, the UGONs are considered potential high-performance T_1_ MRI contrast agents because of their enhanced longitudinal water proton spin relaxivity (r_1_) values, which are higher than those [29] of commercial Gd-chelates and their r_2_/r_1_ ratios, which are close to one (r_2_ = transverse water proton spin relaxivity) [30,31,32,33,34]. Under these conditions, they can provide very high positive (brighter or T_1_) contrast enhancements [30,31,32,33,34]. In addition, they are excretable via the renal system because of their ultrasmall particle size (<3 nm) [35,36], rendering them valuable for in vivo tumor imaging.

Here, enhanced tumor imaging using GlcN as a conjugating ligand of UGONs was investigated. Considering that GlcN alone will not be sufficient in providing colloidal stability for the UGONs because D-glucuronic acid with a similar molecular structure as GlcN did not provide a sufficient colloidal stability such that the D-glucuronic acid-coated nanoparticles settled down in a week, but redispersed via hand-shaking [37], hydrophilic and biocompatible polyacrylic acid (PAA) (Mw = ~1800 Da) [38] was grafted onto the UGONs to obtain PAA-coated UGONs (PAA-UGONs) because PAA with approximately 25 –COOH groups per monomer can provide an excellent colloidal stability [30]. Thereafter, the –COOH groups of PAA were conjugated with many GlcNs via an amide bond to form GlcN-PAA-UGONs. To demonstrate the enhanced tumor imaging of GlcN-PAA-UGONs in T_1_ MRI via GlcN effects, contrast enhancements at various organs including the tumor site were measured prior to and after the intravenous injection of GlcN-PAA-UGONs into human brain glioma (U87MG) tumor-bearing mice tail veins, and the results were compared with those obtained with PAA-UGONs as control.

## 2. Results

### 2.1. Physicochemical Properties of PAA-UGONs and GlcN-PAA-UGONs

**Figure 1:**
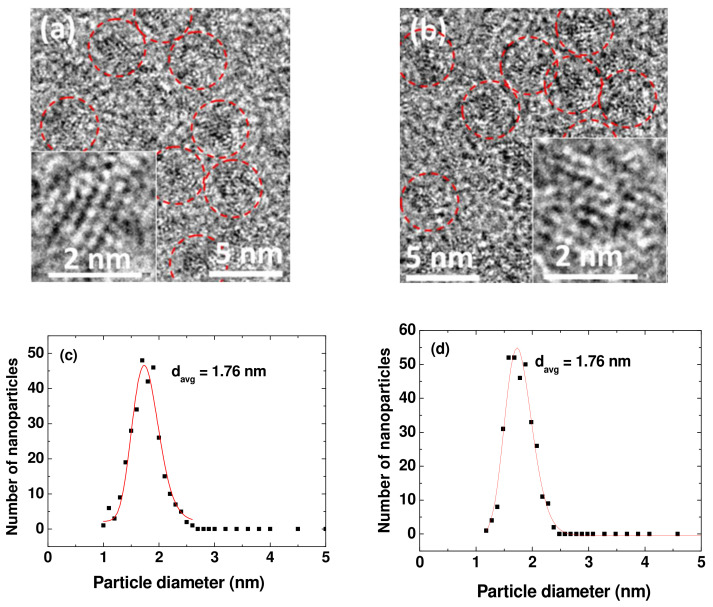

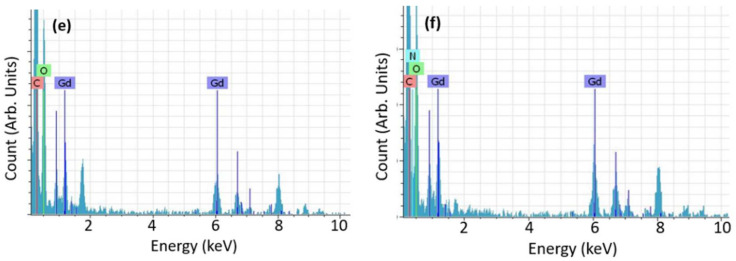
HRTEM images of the (**a**) PAA-UGONs and (**b**) GlcN-PAA-UGONs. Dotted circles indicate nanoparticles, and the insets show magnified HRTEM images on a 2 nm scale. Log-normal function fits to observed particle diameter distributions in the (**c**) PAA-UGONs and (**d**) GlcN-PAA-UGONs. EDS spectra of the (**e**) PAA-UGONs and (**f**) GlcN-PAA-UGONs.

As shown in the high-resolution transmission electron microscopy (HRTEM) images (Figure 1a,b), the particle diameters of the PAA-UGONs and GlcN-PAA-UGONs ranged from 1.0 to 3.0 nm. The insets show the magnified HRTEM images on a 2 nm scale. The average particle diameter (d_avg_) was estimated to be 1.76 nm for the PAA-UGONs and GlcN-PAA-UGONs from log-normal function fits to the observed particle diameter distributions (Figure 1c,d and Table 1). Energy-dispersive X-ray spectroscopy (EDS) confirmed the presence of C, O, and Gd in the PAA-UGONs (Figure 1e) and C, O, N, and Gd in the GlcN-PAA-UGONs (Figure 1f).

**Figure 2:**
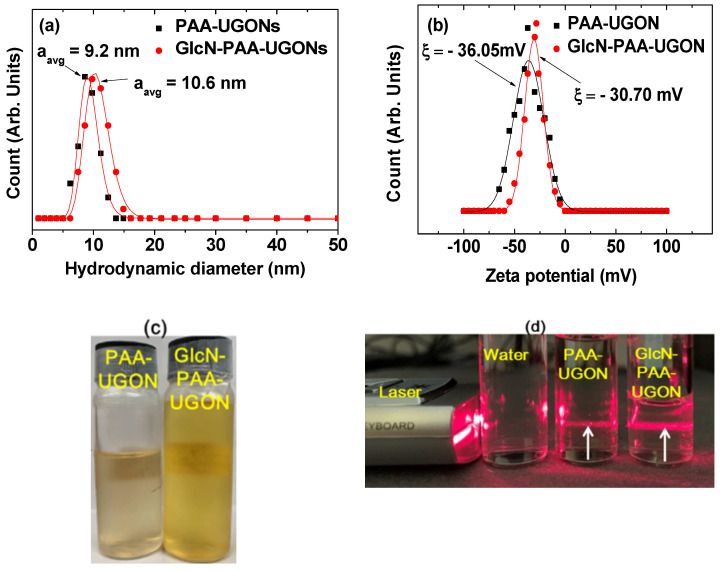
(**a**) DLS patterns of the PAA-UGONs and GlcN-PAA-UGONs in aqueous media and the log-normal function fits to the observed DLS patterns to estimate the a_avg_ values. (**b**) Zeta potential curves of the PAA-UGONs and GlcN-PAA-UGONs in aqueous media. (**c**) Photographs of the PAA-UGONs (left) and GlcN-PAA-UGONs (right) in aqueous media, exhibiting excellent colloidal stability without nanoparticle precipitation after synthesis (>1 year). (**d**) Tyndall effect (light scattering by colloids), confirming the colloidal dispersion of the PAA-UGONs (middle vial) and GlcN-PAA-UGONs (right vial) in aqueous media, whereas no such light scattering was observed in the triple-distilled water (left vial). Arrows indicate laser light scattering by nanoparticle colloids.

Hydrodynamic diameters were determined using dynamic light scattering (DLS) patterns (Figure 2a). The average hydrodynamic diameters (a_avg_) were estimated to be 9.2 and 10.6 nm from the log-normal function fits to the observed hydrodynamic diameter distributions of the PAA-UGONs and GlcN-PAA-UGONs, respectively (Figure 2a and Table 1). The a_avg_ values are larger than the d_avg_ values because of hydrophilic surface coating and accompanying hydration by numerous water molecules around nanoparticles. The a_avg_ of the GlcN-PAA-UGONs was slightly larger than that of the PAA-UGONs because of additional GlcN coating in the GlcN-PAA-UGONs. These large a_avg_ values in both nanoparticles suggested that the nanoparticles attracted a large number of water molecules around them via the hydrophilic –COOH groups of PAA in the PAA-UGONs and via the hydrophilic –OH groups of GlcN and –COOH groups of PAA in the GlcN-PAA-UGONs. This explains the observed excellent colloidal stability in aqueous media; the nanoparticle colloids never settled down after the synthesis (>1 year). High zeta potentials (ξ) were observed for the PAA-UGONs and GlcN-PAA-UGONs in aqueous media, i.e., −36.0 and −30.7 mV, respectively (Figure 2b and Table 1), confirming the observed excellent colloidal stability of the nanoparticles, as observed in similar nanoparticles grafted with hydrophilic polymers [31,39]. The zeta potential of the GlcN-PAA-UGONs was slightly lower than that of the PAA-UGONs. This was probably because the –OH groups of GlcN in the GlcN-PAA-UGONs were less negative than the –COOH group of PAA in the PAA-UGONs. The aqueous nanoparticle solution samples of the PAA-UGONs and GlcN-PAA-UGONs are shown in Figure 2c, exhibiting transparency, owing to the excellent colloidal stability of the samples. The Tyndall effect (light scattering by colloids) was observed only for the solution samples (middle vial for the PAA-UGONs and right vial for the GlcN-PAA-UGONs, as shown in Figure 2d), but not for the triple-distilled water (left vial, as shown in Figure 2d), confirming the colloidal dispersions in aqueous media.

### 2.2. Crystal Structures

**Figure 3:**
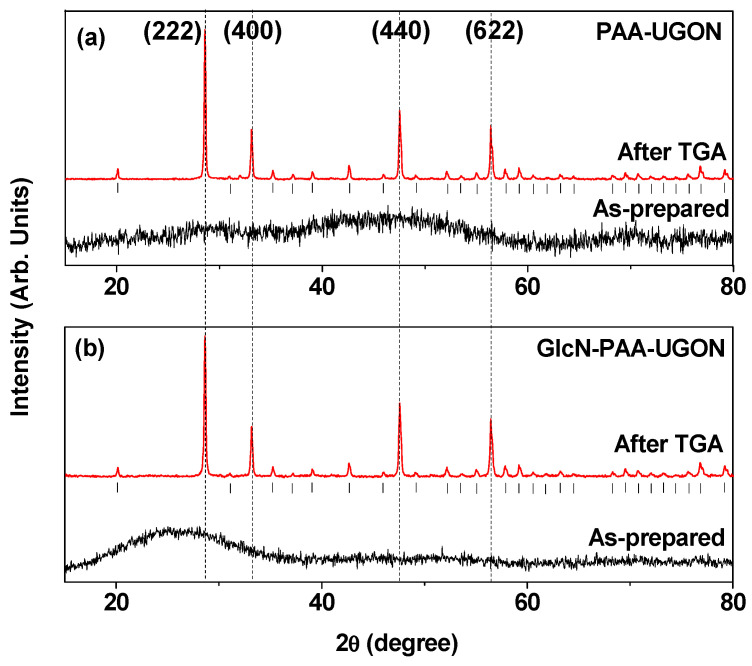
XRD patterns prior to (as-prepared) and after TGA: (**a**) PAA-UGONs and (**b**) GlcN-PAA-UGONs. The representative assignments on strong XRD peaks after TGA are (*hkl*) Miller indices of cubic Gd_2_O_3_. All peaks (labeled with vertical bars below the XRD peaks after TGA) could be designated (*hkl*) Miller indices of cubic Gd_2_O_3_.

X-ray diffraction (XRD) patterns of the synthesized nanoparticle powder samples were recorded prior to and after thermogravimetric analysis (TGA) (Figure 3). The XRD patterns prior to the TGA did not exhibit sharp peaks because the nanoparticles were not fully crystallized, owing to their ultrasmall particle sizes. However, the XRD patterns obtained after TGA exhibited sharp peaks of body-centered cubic (bcc) Gd_2_O_3_ [40]. This was attributed to both particle size and crystal growth during the TGA up to 900 °C. The lattice constant of the TGA-treated powder samples was estimated to be 10.814 Å, which were consistent with a reported value (10.813 Å) [40].

### 2.3. Surface-Coating Results

**Figure 4:**
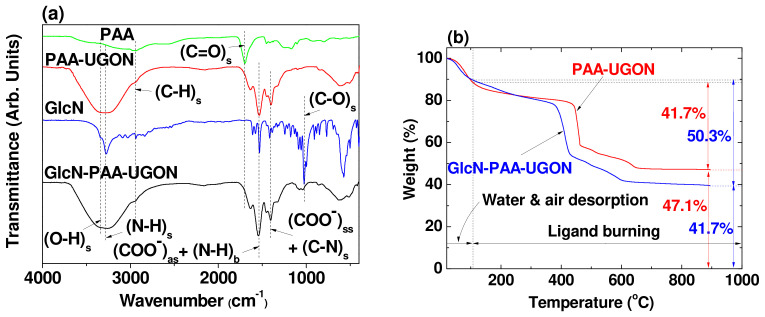
(**a**) FT-IR absorption spectra of PAA, GlcN, PAA-UGONs, and GlcN-PAA-UGONs. Subscripts as, ss, s, and b indicate asymmetric stretching, symmetric stretching, stretching, and bending, respectively. (**b**) TGA curves of the PAA-UGONs and GlcN-PAA-UGONs.

The surface coating of the UGONs with PAA and the successful conjugation of GlcN with PAA in the PAA-UGONs via the amide bond were confirmed using the Fourier transform infrared (FT-IR) absorption spectra of PAA, GlcN, PAA-UGONs, and GlcN-PAA-UGONs (Figure 4a). The C=O stretching vibration at 1700 cm^−1^ in PAA was red-shifted and split into two peaks in the PAA-UGONs and GlcN-PAA-UGONs (COO^−^ antisymmetric stretching vibration at 1538 cm^−1^ and COO^−^ symmetric stretching at 1401 cm^−1^), confirming the successful coating of PAA on the UGON surface. This red-shift was due to the hard acid–hard base type of bonding between the COO^−^ group of PAA (hard base) and Gd^3+^ of the UGONs (hard acid) [41,42,43], and the splitting was due to the bridge-bonding between the COO^−^ group and Gd^3+^ [44]. The C–H stretching vibration at 2957 cm^−1^ in PAA appeared at 2945 cm^−1^ in the PAA-UGONs and GlcN-PAA-UGONs, confirming the surface coating of UGON with PAA again. The N–H bending and C–N stretching vibrations at 1540 and 1415 cm^−1^, respectively, in GlcN overlapped with the COO^−^ antisymmetric and symmetric stretching vibrations in the GlcN-PAA-UGONs. The C–O stretching vibration at 1026 cm^−1^ in GlcN appeared at 1060 cm^−1^ in the GlcN-PAA-UGONs. The observed FT-IR absorption frequencies were consistent with those in previous studies [30,45,46,47], and are summarized in Table 2.

The surface-coating amounts (P) of PAA in the PAA-UGONs and GlcN-PAA in the GlcN-PAA-UGONs were measured via TGA and elemental analysis (EA). As shown in the TGA curves (Figure 4b), the surface-coating amounts in wt.% were estimated to be 41.7 and 50.3% for the PAA-UGONs and GlcN-PAA-UGONs, respectively, from the mass drops after considering the initial mass drops between room temperature and ~105 °C, owing to water and air desorption (Table 1). The remaining masses were due to the UGONs (Figure 4b and Table 1). The difference in the coating amount (8.6%) between the PAA-UGONs and GlcN-PAA-UGONs was due to the GlcN in the GlcN-PAA-UGONs. For EA, the surface-coating amounts in wt.% were estimated to be 48.2 (C/H/O = 20.83/3.18/24.14 in wt.%) for the PAA-UGONs and 54.4 (C/H/O/N = 23.33/4.04/25.25/1.73 in wt.%) for the GlcN-PAA-UGONs by summing the wt.% of C, H, O, and N. The difference (6.2%) was due to the GlcN in the GlcN-PAA-UGONs, as previously mentioned. The higher *p* values of EA, compared with those of TGA, were due to the water and air contributions to the P in the EA because all elements in the sample except for UGONs were measured during the EA. Using the TGA data, the grafting density (σ) of the PAA-UGONs, corresponding to the average number of PAA polymers coating a UGON unit surface area [48], was estimated to be 0.64 nm^−2^ using the bulk density of Gd_2_O_3_ (7.407 g/cm^3^) [49], *p* value previously estimated, and d_avg_ determined from the HRTEM imaging. By multiplying σ by the UGON surface area (πd_avg_^2^), the average number (N_NP_) of PAA polymers per UGON was estimated to be ~ 6. To estimate the number of GlcN molecules per UGON in the GlcN-PAA-UGONs, the molecular weight of GlcN-PAA increased in the GlcN unit until the N_NP_ was 6 or σ was 0.64 because the N_NP_ and σ of PAA in the GlcN-PAA-UGONs should be the same as those in the PAA-UGONs. Approximately, five GlcN molecules per PAA were conjugated. Thus, ~30 GlcN molecules per UGON were conjugated. The surface-coating results are summarized in Table 1.

### 2.4. Magnetic Properties

**Figure 5:**
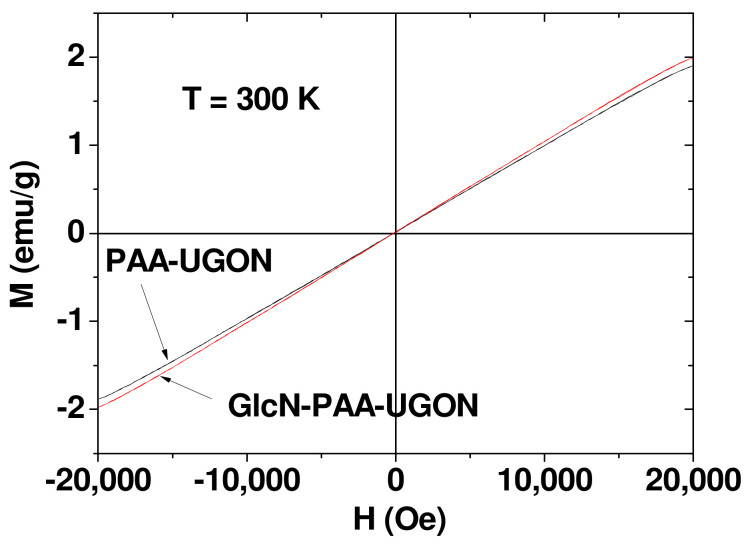
Mass-corrected M–H curves for the PAA-UGONs and GlcN-PAA-UGONs at 300 K using net masses of UGONs estimated from the TGA curves (only UGONs without surface-coating ligands).

The magnetic properties of the UGONs were investigated by measuring the magnetization (M) versus applied field (H) (or M–H) curves (−2.0 T ≤ H ≤ 2.0 T) at 300 K using a vibrating sample magnetometer (VSM) (Figure 5). The measured M values were mass-corrected using net masses of UGONs without ligands, which were obtained from the net masses of UGONs in the TGA curves. As shown in Figure 5, all nanoparticle samples were paramagnetic, exhibiting no hysteresis, small unsaturated M values, zero remanence, and zero coercivity, similar to bulk Gd_2_O_3_ [50,51]. From the mass-corrected M–H curves, the unsaturated net M values of the UGONs at H = 2.0 T were estimated to be 1.89 and 1.98 emu/g for the PAA-UGONs and GlcN-PAA-UGONs, respectively (Table 3). Therefore, the average M value for the UGONs was 1.94 emu/g. This appreciable M value at room temperature was due to a high spin magnetic moment (s = 7/2) of Gd^3+^ [29] and was attributable to high r_1_ values of Gd^3+^-based MRI contrast agents [29,30,31,32,33,34].

### 2.5. r_1_ and r_2_ Values and R_1_ and R_2_ Map Images

**Figure 6:**
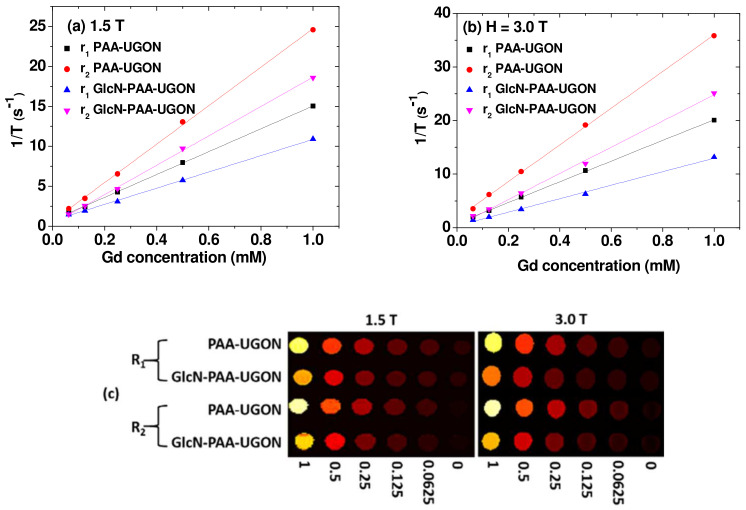
Plots of 1/T_1_ and 1/T_2_ as a function of the Gd concentration for the PAA-UGONs and GlcN-PAA-UGONs in aqueous media at H = (**a**) 1.5 and (**b**) 3.0 T. The slopes corresponded to the r_1_ and r_2_ values, respectively. (**c**) R_1_ and R_2_ map images showing dose-dependent contrast enhancements.

The r_1_ and r_2_ values and longitudinal (R_1_) and transverse (R_2_) map images were measured at H = 1.5 and 3.0 T MR fields; the r_1_ and r_2_ values were estimated from the plots of inverse longitudinal (T_1_) and transverse (T_2_) water proton spin relaxation times, 1/T_1_ and 1/T_2_ as a function of the Gd concentration, respectively (Figure 6a,b and Table 3). The r_1_ and r_2_ values increased with an increase in H from 1.5 to 3.0 T (Table 3). This was because they were proportional to M^2^ [52,53], and M increased with an increase in H because the UGONs were paramagnetic (refer to M–H curves in Figure 5). As shown in Figure 6c, dose-dependent contrast enhancements were observed in the R_1_ and R_2_ map images for both samples in aqueous media. This demonstrated in vitro that both samples induced T_1_ and T_2_ water proton spin relaxations. However, both nanoparticles were more suitable as T_1_ MRI contrast agents rather than as T_2_ MRI contrast agents because their r_2_/r_1_ ratios were close to one, and their r_1_ values were extremely high (3 to 4 times higher than those [29] of commercial Gd-chelates).

### 2.6. In Vitro Cellular Cytotoxicity Results

**Figure 7:**
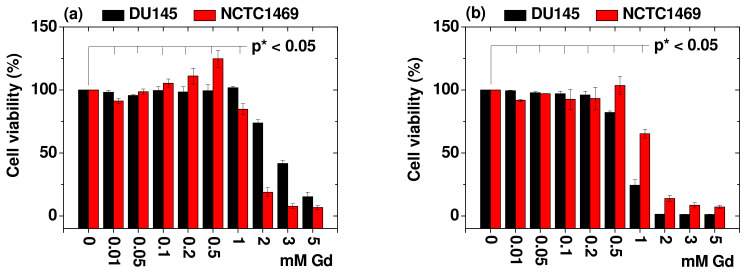

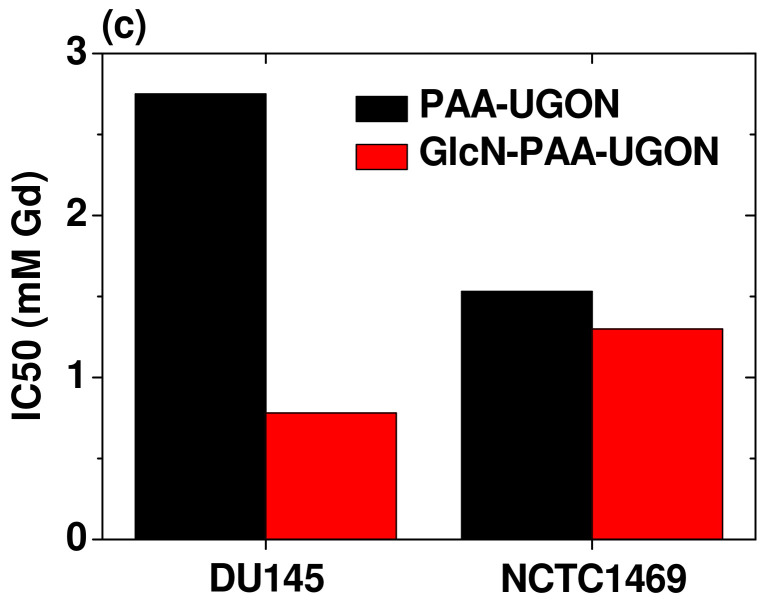
Plots of cell viabilities of the (**a**) PAA-UGONs and (**b**) GlcN-PAA-UGONs in DU145 and NCTC1469 cell lines. The significance of the results was confirmed by a student *t*-test (*p* * < 0.05 was considered statistically important). (**c**) Plots of IC50 of PAA-UGONs and GlcN-PAA-UGONs in DU145 and NCTC1469 cells.

The biocompatibility of the PAA-UGONs and GlcN-PAA-UGONs was investigated by measuring the in vitro cell viabilities in normal mouse hepatocyte (NCTC1469) and human prostate tumor (DU145) cell lines (Figure 7). The cytotoxicity results up to 0.5 mM Gd are consistent with those measured in PAA-coated lanthanide oxide nanoparticles [39,54], indicating the suitability of PAA as an excellent biocompatible surface-coating ligand. The IC50 (the inhibitory concentration of chemicals that cause 50% of the maximum inhibition) of the PAA-UGONs and GlcN-PAA-UGONs were estimated to be 2.75 and 0.78 mM Gd in DU145 cell lines, respectively, and 1.53 and 1.30 mM Gd in NCTC1469 cell lines, respectively (Figure 7c). These values are more or less consistent with 1.93 mM (or 304 μg/mL) of gadolinium oxide nanoparticles prepared via thermal decomposition method in human umbilical vein endothelial (HUVEC) cells [55].

### 2.7. In Vivo Tumor Imaging: T_1_ MR Images in U87MG Tumor-Bearing Mice

**Figure 8:**
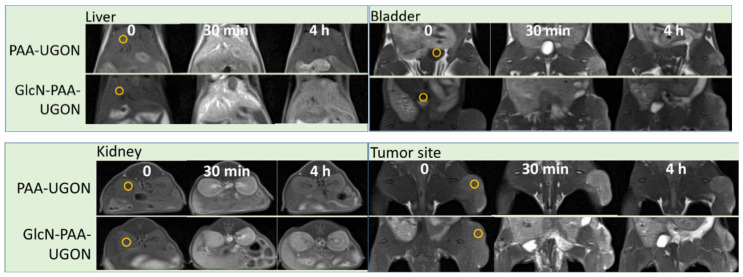
T_1_ MR images of U87MG tumor-bearing mice prior to (indicated as “0”) and after the intravenous injection of the GlcN-PAA-UGONs and PAA-UGONs as control into the tail veins. Small circles prior to injection indicate regions-of-interest (ROIs) used to estimate signal-to-noise ratios (SNRs).

In vivo T_1_ MR images of the U87MG tumor-bearing mice were obtained prior to (labeled as “0”) and after the intravenous injection of aqueous solution samples of the GlcN-PAA-UGONs and PAA-UGONs as control into the tail veins (Figure 8). As shown in Figure 8, positive contrast enhancements were observed after the injection in various organs (liver, kidney, and bladder), including the tumor site, for all samples. However, the contrast enhancements of the GlcN-PAA-UGONs were higher than those of the PAA-UGONs, except for the bladder because the PAA-UGONs were rapidly excreted through the renal system within ~4 h after injection. In addition, the contrast enhancements of the GlcN-PAA-UGONs were retained longer in all organs, including the tumor site, compared with those of the PAA-UGONs. These results were attributable to the GlcN effects in the GlcN-PAA-UGONs and are quantitatively discussed in the following section, by measuring the signal-to-noise ratios (SNRs) of regions of interest (ROIs) in T_1_ MR images.

## 3. Discussion

**Figure 9:**
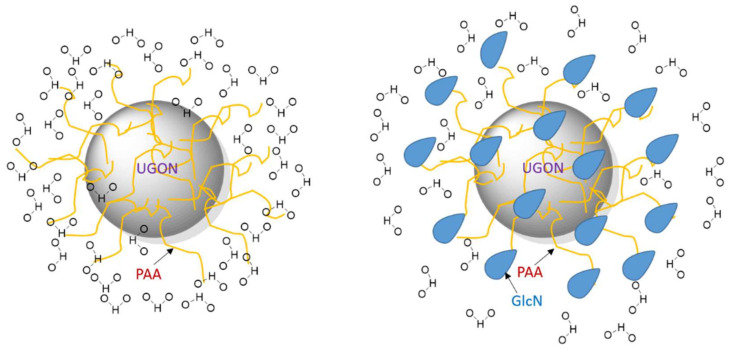
Schematic diagrams showing closer and denser water molecules around PAA-UGONs (**left**), compared with those around GlcN-PAA-UGONs (**right**). Both Chemdraw and Powerpoint were used to draw figures.

The r_1_ and r_2_ values of the GlcN-PAA-UGONs were slightly lower than those of the PAA-UGONs (Table 3). This was probably because the PAA with many –COOH groups attracted a larger number of water molecules around the UGONs than GlcN with –OH groups. In addition, the hydrodynamic diameter of the GlcN-PAA-UGONs was slightly larger than that of the PAA-UGONs (Table 1), owing to the extra GlcN coating in the GlcN-PAA-UGONs as previously mentioned, rendering the water molecules in the GlcN-PAA-UGONs slightly farther apart from the UGONs than those in the PAA-UGONs. Based on these, hypothesized distributions of water molecules around the nanoparticle are drawn in Figure 9. The first case allows the GlcN-PAA-UGONs to interact with a fewer number of water proton spins than the PAA-UGONs, and the second case allows the GlcN-PAA-UGONs to less strongly interact with water proton spins than the PAA-UGONs, resulting in lower r_1_ and r_2_ values in the GlcN-PAA-UGONs.

**Figure 10:**
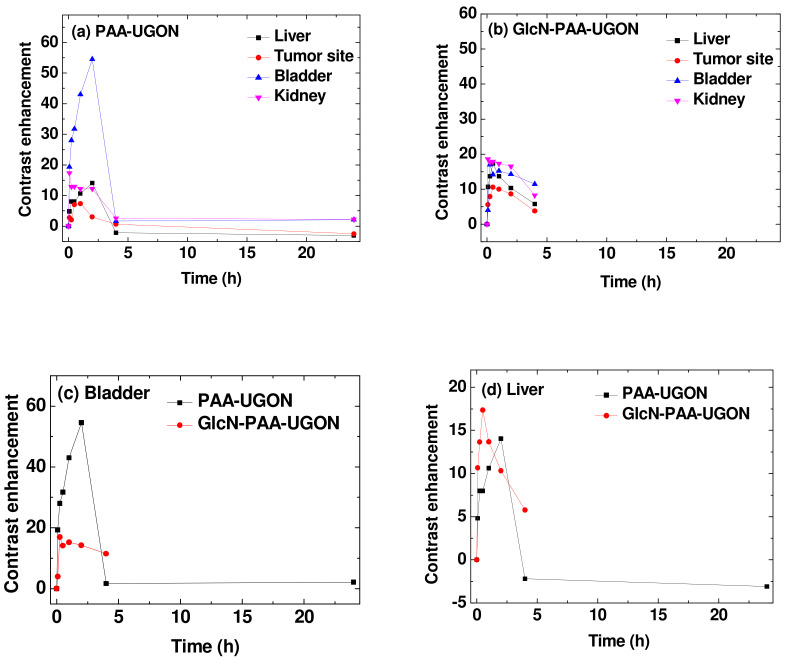

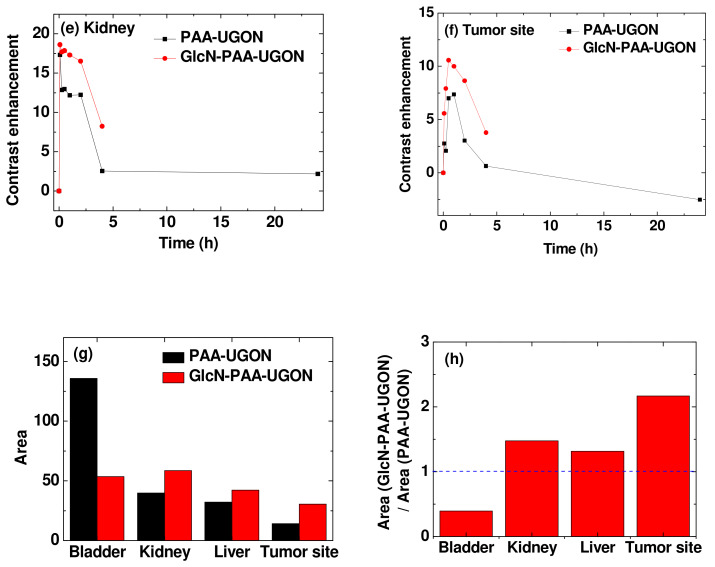
Plots of contrast enhancements [SNR-ROI (time)–SNR-ROI (0)] as a function of time for the (**a**) PAA-UGONs and (**b**) GlcN-PAA-UGONs. Plots of contrast enhancements as a function of time for the PAA-UGONs and GlcN-PAA-UGONs in the (**c**) bladder, (**d**) liver, (**e**) kidney, and (**f**) tumor site. (**g**) Plots of contrast enhancement curve areas below the curves between 0 and 4 h in the liver, kidney, bladder, and tumor site. (**h**) Plots of area ratios for the GlcN-PAA-UGONs to the PAA-UGONs in the liver, kidney, bladder, and tumor site.

**Figure 11:**
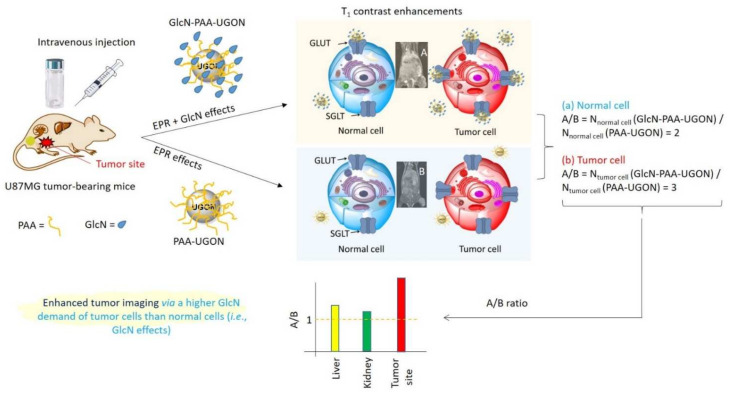
Schematic illustration of contrast enhancements in the tumor site via enhanced permeability and retention (EPR) and GlcN effects. The PAA-UGONs allow only the EPR effects whereas the GlcN-PAA-UGONs allow the EPR and GlcN effects. Consequently, GlcN-PAA-UGONs provide the enhanced tumor imaging via the additional GlcN effects, compared with PAA-UGONs.

To quantitatively determine the enhanced tumor imaging of GlcN-PAA-UGONs via GlcN effects, the SNRs of ROIs (labeled as small circles in the T_1_ MR images prior to injection, as shown in Figure 8) at the tumor site, liver, kidney, and bladder were estimated and subtracted from those prior to injection to estimate the contrast enhancements [=SNR-ROI (time) − SNR-ROI (0)]. The contrast enhancements were plotted as a function of time in Figure 10a,b for the PAA-UGONs and GlcN-PAA-UGONs, respectively. As shown in Figure 10a, most of the PAA-UGONs were excreted via the renal system within 4 h after injection because of their ultrasmall particle sizes, which can be noticed from considerably higher contrast enhancements in the bladder, compared with those in other organs, including the tumor site. However, the GlcN-PAA-UGONs exhibited no such drastic contrast enhancements in the bladder (Figure 10b), indicating their slower excretion through the renal system, compared with PAA-UGONs, owing to their uptakes by GLUT and SGLT transporters expressed on the normal and tumor cell membranes [13,14,15]. Consequently, apart from the bladder (Figure 10c), the contrast enhancements of the GlcN-PAA-UGONs in the liver (Figure 10d), kidney (Figure 10e), and tumor site (Figure 10f) were higher than those of the PAA-UGONs. For a quantitative comparison between the PAA-UGONs and GlcN-PAA-UGONs in each organ and tumor site, the areas below the curves between 0 and 4 h are plotted in Figure 10g. Higher areas below the curves for the GlcN-PAA-UGONs, compared with those of the PAA-UGONs in each organ and the tumor site except for the bladder were observed, implying higher accumulations of GlcN-PAA-UGONs than PAA-UGONs in these organs and tumor site, owing to the aforementioned reason. Larger areas of organs (liver and kidney) below the curves than those of the tumor site for the PAA-UGONs and GlcN-PAA-UGONs were probably because the nanoparticles were excreted through these organs. Importantly, the area ratios of the GlcN-PAA-UGONs to the PAA-UGONs (Figure 10h) was the highest at the tumor site, implying the highest accumulations of the GlcN-PAA-UGONs with respect to those of PAA-UGONs at the tumor site, which is due to GlcN effects. It is well-known that tumor cells grow via angiogenesis after tumor cell inoculation into mice [56,57] and that nanoparticles accumulate at tumor site via EPR effects [5,6,8], thus enhancing T_1_ contrasts in MR images. The EPR effects are the same for the GlcN-PAA-UGONs and PAA-UGONs, but the GlcN-PAA-UGONs have the additional GlcN effects. Using these two effects, the observed contrast enhancements are schematically illustrated in Figure 11 using the number (N) for the UGONs. As shown in Figure 11, the N_tumor_ _cell_ (GlcN-PAA-UGONs) > N_normal cell_ (GlcN-PAA-UGONs) is due to GlcN and EPR effects and the N_tumor_ _cell_ (PAA-UGONs) > N_normal cell_ (PAA-UGONs) is due to EPR effects. As the contrast enhancement is proportional to N, the observed highest contrast enhancements in the tumor site (Figure 10h) can be explained as due to GlcN effects. Therefore, the GlcN-PAA-UGONs can highly enhance contrasts in tumor site via the additional GlcN effects. GlcN is not tumor-specific; therefore, GlcN-PAA-UGONs can be applied to any tumor type imaging. As shown in Figure 7c, the GlcN-PAA-UGONs exhibited lower IC50 values than the PAA-UGONs for the same cell lines, indicating higher toxicities than PAA-UGONs for the same cell lines, likely due to higher accumulation of GlcN-PAA-UGONs in tumor DU145 and normal NCTC1469 cells than PAA-UGONs because of GlcN. Notably, IC50 value of the GlcN-PAA-UGONs in DU145 cells was the lowest among four IC50 values. Assuming that the cellular toxicity is proportional to the number (N) of UGONs accumulated into cells (i.e., N∝1/IC50), N_DU145_ (GlcN-PAA-UGONs)/N_DU145_ (PAA-UGONs) = 3.53 > N_NCTC1469_ (GlcN-PAA-UGONs)/N_NCTC1469_ (PAA-UGONs) = 1.18, also explaining the GlcN effects.

## 4. Materials and Methods

### 4.1. Chemicals

GdCl_3_∙6H_2_O (99.9%), NaOH (>99.9%), triethylene glycol (TEG) (99%), PAA (Mn = ~1800 Da), D-glucosamine-hydrochloride (>99%), N-hydroxysuccinimide (NHS) (98%), 1-ethyl-3 (3-dimethylaminopropyl) carbodiimide (EDC) (97%), and dialysis tube [molecular weight cut-off (MWCO) = ~2000 Da] were purchased from Sigma-Aldrich, St. Louis, MO, USA and used as received. Ethanol (>99%) was purchased from Duksan, South Korea, and used as received for the initial washing of the nanoparticles. Triple-distilled water was used for the final washing of the nanoparticles and the preparation of nanoparticle solution samples.

### 4.2. One-Pot Polyol Synthesis of the PAA-UGONs

**Figure 12:**
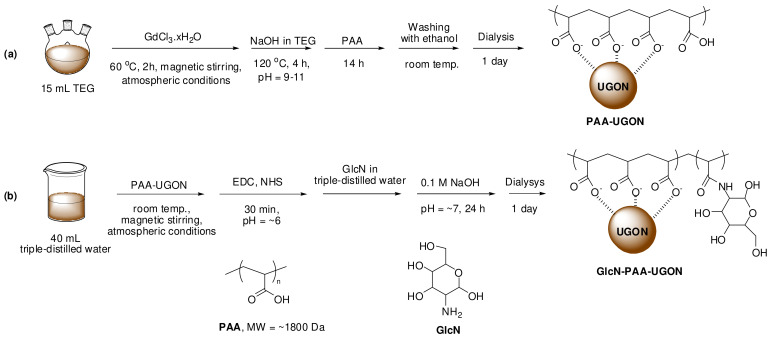
(**a**) One-pot polyol synthesis of PAA-UGONs and (**b**) conjugation of the –NH_2_ group of GlcN with the –COOH group of PAA in the PAA-UGONs via an amide bond to obtain the GlcN-PAA-UGONs.

The PAA-UGONs were synthesized using a simple one-pot polyol method (Figure 12a). First, 2 mmol of GdCl_3_∙6H_2_O was added to 15 mL of TEG in a three-necked round-bottom flask, and the mixture solution was magnetically stirred at 60 °C under atmospheric conditions until the precursor dissolved in TEG (it lasted for ~2 h). In a separate beaker, 10 mmol of NaOH in 10 mL of TEG was prepared. The NaOH solution was added to the aforementioned precursor solution until the pH of the solution was within a range of 9–11. The reaction temperature increased to 120 °C, and the mixture solution was magnetically stirred for 4 h. Thereafter, 0.25 mmol of PAA was added to the reaction solution with magnetic stirring for 14 h. The product solution containing the PAA-UGONs was cooled to room temperature and transferred into a 500 mL beaker. Subsequently, 400 mL of ethanol was added to the product solution with magnetic stirring for 10 min. The product solution was preserved in a refrigerator until the PAA-UGONs settled to the beaker bottom. The top transparent solution was removed, and the remaining product solution was washed again with ethanol using the same process thrice. The product solution was dialyzed (MWCO = ~2000 Da) against 1.5 L of triple-distilled water for one day to remove the remaining impurities, such as Gd^3+^, Cl^−^, Na^+^, TEG, ethanol, and PAA from the product solution.

### 4.3. Synthesis of the GlcN-PAA-UGONs

The –NH_2_ group of GlcN was conjugated with the –COOH group of PAA in the PAA-UGONs via the EDC/NHS coupling method (Figure 12b). The prepared PAA-UGONs were dispersed in 40 mL of triple-distilled water in a 250 mL beaker. Thereafter, 1 mmol of EDC and 2 mmol of NHS were added to the aforementioned solution at room temperature under atmospheric conditions with magnetic stirring for 30 min (the solution pH was ~6). Afterward, 10 mmol of GlcN prepared in 4 mL of triple-distilled water was added to the solution with magnetic stirring for 20 min. Subsequently, a 0.1 M NaOH solution was slowly added to the solution to obtain a solution pH of ~7. The solution was magnetically stirred for 24 h. The product solution containing the GlcN-PAA-UGONs was dialyzed (MWCO = ~2000 Da) against 1.5 L of triple-distilled water for one day to remove the remaining impurities, such as Na^+^, Cl^−^, EDC, NHS, and GlcN from the product solution.

### 4.4. Physicochemical Property Characterization

The nanoparticle diameter was measured using an HRTEM (Titan G2 ChemiSTEM CS Probe; FEI, Hillsboro, OR, USA) at 200 kV acceleration voltage. For the measurements, a drop of the diluted colloidal nanoparticle sample dispersed in ethanol was dropped onto a carbon film supported by a 200-mesh copper grid (Pelco No. 160, Ted Pella Inc., Redding, CA, USA) using a micropipette (2–20 μL, Eppendorf, Hamburg, Germany) and allowed to dry in air at room temperature. The copper grid with the nanoparticle sample was placed inside the HRTEM for measurements. An EDS instrument (Quantax Nano, Bruker, Berlin, Germany) installed in the HRTEM was used to analyze elements (C, N, O, and Gd) in the nanoparticle samples. The Gd concentration of the nanoparticle samples in aqueous media was determined via inductively coupled plasma-atomic emission spectroscopy (ICP-AES) (IRIS/AP, Thermo Jarrell Ash Co., Waltham, MA, USA). A DLS particle size analyzer (Zetasizer Nano ZS, Malvern, Malvern, UK) was used to measure the hydrodynamic diameter (a) of the nanoparticle colloids in aqueous media (0.01 mM Gd). The zeta potentials of the nanoparticle colloids in aqueous media (0.01 mM Gd) were measured using the same DLS instrument. A multipurpose XRD (MP-XRD) machine (X’PERT PRO MRD, Philips, Amsterdam, The Netherlands) with unfiltered CuKa (λ = 0.154184 nm) radiation was used to characterize the crystal structures of the nanoparticle powder samples. The scanning step and scan range in 2θ were 0.033 and 15–100°, respectively. The attachment of PAA to the UGONs and the conjugation of GlcN with the PAA in the PAA-UGONs were investigated by recording the FT-IR absorption spectra (Galaxy 7020A, Mattson Instrument Inc., Madison, WI, USA) using powder samples pelletized with KBr. The scan range was 400–4000 cm^−1^. A TGA instrument (SDT-Q600, TA Instrument, New Castle, DE, USA) was used to estimate the surface-coating amount by recording TGA curves between room temperature and 900 °C under air flow. The average amounts of the surface-coated PAA and GlcN-PAA in wt.% were estimated from the initial mass losses after considering the water and air desorption between room temperature and ~105 °C. The amounts of UGONs were estimated from the remaining masses. After the TGA, the powder samples were collected and subjected to XRD for identification. EA (Flash 2000, Thermo Fischer, Waltham, MA, USA) was performed using the powder samples to investigate the surface-coating composition (C, H, O, N) and the surface-coating amount in wt.% by summing all elemental wt.%. A VSM (7407-S, Lake Shore Cryotronics Inc., Westerville, OH, USA) was used to characterize the magnetic properties of the nanoparticle powder samples by recording M–H curves (−2.0 T ≤ H ≤ 2.0 T) at 300 K. The measurements were performed using powder samples of 20–30 mg. The net M value of each sample (only the UGONs without the PAA and GlcN-PAA coating) was estimated using the net mass of the UGONs extracted from the TGA curve.

### 4.5. In Vitro Cellular Cytotoxicity Measurements

The in vitro cellular cytotoxicity of the aqueous nanoparticle suspension samples was measured using a CellTiter-Glo Luminescent Cell Viability Assay (Promega, Madison, WI, USA). The intracellular adenosine triphosphate was quantified using a Victor 3 luminometer (Perkin Elmer, Waltham, MA, USA). Two cell lines, NCTC1469 and DU145, were used. Each cell line was seeded onto a separate 24-well cell culture plate and incubated for 24 h (5 × 10^4^ cell density, 500 μL cells/well, 5% CO_2_, and 37 °C). Nine test solutions (0.01, 0.05, 0.1, 0.2, 0.5, 1, 2, 3, 5 mM Gd) were prepared by diluting the original concentrated nanoparticle suspension sample dispersed in triple-distilled water with a sterile phosphate-buffered saline (PBS) solution. Afterward, 2 μL aliquots were used to treat the cells, which were subsequently incubated for 48 h. Cell viabilities were measured thrice to obtain average cell viabilities, which were normalized with respect to that of the untreated control cells (0.0 mM Gd).

### 4.6. Water Proton Spin Relaxivity and Map Image Measurements

The T_1_ and T_2_ water proton spin relaxation times and the R_1_ and R_2_ map images were measured using a 1.5 T MRI scanner (GE 1.5 T Signa Advantage, GE Medical Systems, Chicago, IL, USA) equipped with a knee coil (EXTREM) and 3.0 T MRI scanner (Magnetom Trio Tim, Siemens, Munich, Bayern, Germany). Aqueous dilute solutions (1, 0.5, 0.25, 0.125, and 0.0625 mM Gd) were prepared by diluting the original concentrated solutions with triple-distilled water. These dilute solutions were used to measure the T_1_ and T_2_ relaxation times and R_1_ and R_2_ map images. The r_1_ and r_2_ water proton spin relaxivities were estimated from the slopes of the plots of 1/T_1_ and 1/T_2_ versus Gd concentration, respectively. T_1_ relaxation time measurements were conducted using an inversion recovery method. In this method, the inversion time (TI) was varied, and the MR images were acquired at 35 different TI values in the range of 50–1750 ms. The T_1_ relaxation times were obtained from the nonlinear least-square fits to the measured signal intensities at various TI values. For the measurements of T_2_ relaxation times, the Carr–Purcell–Meiboom–Gill pulse sequence was used for multiple spin-echo measurements, and 34 images were acquired at 34 different echo time (TE) values in the range of 10–1900 ms. The T_2_ relaxation times were obtained from the nonlinear least-square fits to the mean pixel values of the multiple spin-echo measurements at various TE values.

### 4.7. Animal Experiments

All in vivo experiments on mice were performed following the rules, regulations, and permission of the animal research committee of the Korea Institute of Radiological and Medical Sciences (approval number: Kirams2018-0072 and approval date: 9 January 2019).

### 4.8. Tumor Model Nude Mice Preparation

The U87MG tumor cells were incubated for 24 h at 37 °C in air containing 5% CO_2_. Roswell Park Memorial Institute (RPMI-1640) containing 10% (*v*/*v*) fetal bovine serum and 1% (*v*/*v*) penicillin streptomycin was used as the culture medium of the cells. BALB/c nude mice (male, 5-week old, 20 g) were administered inoculation into their subcutaneous tissue in one of their hind legs (thighs) with 5 × 10^6^ U87MG tumor cells suspended in 100 μL of PBS solution. In vivo MRI experiments were conducted three weeks after the tumor cell inoculation.

### 4.9. In Vivo T_1_ MR Image Measurements

In vivo T_1_ MR images were acquired using a 3.0 T MRI scanner (Magnetom Trio Tim, Siemens, Munich, Bayern, Germany). For imaging, U87MG tumor-bearing mice were anesthetized with 1.5% isoflurane in oxygen. The aqueous nanoparticle suspension samples (PAA-UGONs and GlcN-PAA-UGONs) were injected into the tail veins of the mice (0.1 mmol Gd/kg) as a bolus: two mice were used for each nanoparticle sample. All mice recovered after injection. Measurements were performed prior to and after the injection. During the measurements, the body temperature of the mice was maintained at 37 °C using a warm water blanket. The spin-echo sequence was used to obtain T_1_ MR images. The typical measurement parameters used for coronal (or axial) image measurements are as follows: H = 3.0 T, TE = 10 (9.3) ms, repetition time = 385 (455) ms, echo train length = 3 (3) mm, pixel bandwidth = 299 (299) Hz, flip angel = 120 (120) degree, width = 41.875 (60) mm, height = 60 (45) mm, number of acquisitions = 8 (4), field of view = 70 (70) mm, slice thickness = 1.0 (1.5) mm, and spacing = 1.1 (3.75) mm. The numbers in parentheses are those used for the axial image measurements.

## 5. Conclusions

Using GlcN effects, the enhanced tumor imaging of GlcN-PAA-UGONs was investigated using tumor model nude mice. The PAA-UGONs were used as control. The results are summarized as follows.

(1) The particle diameter was ultrasmall (average diameter = 1.76 nm).
(2) The PAA-UGONs and GlcN-PAA-UGONs exhibited excellent colloidal stability in aqueous media (no precipitation after synthesis, >1 year) and low cellular toxicities up to 0.5 mM Gd.
(3) The PAA-UGONs and GlcN-PAA-UGONs exhibited three to four times higher r_1_ values than those of commercial Gd-chelates, and their r_2_/r_1_ ratios were close to one, indicating that they are potential high-performance T_1_ MRI contrast agents.
(4) The GlcN-PAA-UGONs exhibited higher contrast enhancements at various organs, including the tumor site, compared with the PAA-UGONs, and such contrast enhancements were the highest at the tumor site, owing to the GlcN effects. Consequently, the GlcN-PAA-UGONs can be applied to highly enhancing contrasts in tumor.

## Figures and Tables

**Table 1:** Physicochemical properties of the PAA-UGONs and GlcN-PAA-UGONs.

Ligand	d_avg_ (nm)	a_avg_ (nm)	ξ (mV)	Surface-Coating Results			
				P ^a^ (wt.%)	UGON (wt.%)	σ ^b^ (l/nm^2^)	N_NP_ ^c^
PAA	1.76 ± 0.01	9.2 ± 0.1	–36.0 ± 0.5	41.7 (48.2)	47.1	0.64	~6 PAA
GlcN-PAA	1.76 ± 0.01	10.6 ± 0.1	–30.7 ± 0.2	50.3 (54.4)	39.3	0.64	~6 (PAA + ~5 GlcN)

^a^ Values in parentheses are estimated from elemental analysis (EA). ^b^ Grafting density, i.e., average number of molecules coating a nanoparticle unit surface area. ^c^ Average number of molecules coating a nanoparticle surface.

**Table 2:** Summary of observed FT-IR absorption frequencies in cm^−1^.

Vibration ^a^	PAA	GlcN	PAA-UGON	GlcN-PAA-UGON	Ref
(C-O) _s_	-	1026	-	1060	[30,45,46]
(C-N) _s_	-	1415	-	1401	[45]
(COO-) _ss_	-	-	1401	1401	[30,45]
(COO-) _as_	-	-	1538	1538	[30,45]
(N-H) _b_	-	1540	-	1538	[46]
(C=O) _s_	1700	-	-	-	[30]
(C-H) _s_	2957	2940	2945	2945	[30,45,46]
(N-H) _s_	-	3283	-	~3319	[45,46,47]
(O-H) _s_	-	3346	~3319	~3319	[45,46,47]

^a^ Subscripts _as_, _ss_, _s_, and _b_ indicate asymmetric stretching, symmetric stretching, stretching, and bending, respectively.

**Table 3:** Magnetic properties and r_1_ and r_2_ values of the PAA-UGONs and GlcN-PAA-UGONs.

Nanoparticle	Magnetic Properties		Water Proton Spin Relaxivities (s^−1^mM^−1^)					
	Magnetism	Net M at 2.0 T (emu/g)	H = 1.5 T			H = 3.0 T		
			r_1_	r_2_	r_2_/r_1_	r_1_	r_2_	r_2_/r_1_
PAA-UGON	Paramagnetic	1.89	14.31	24.09	1.68	19.32	34.23	1.77
GlcN-PAA-UGON	Paramagnetic	1.98	10.18	18.38	1.80	12.60	24.46	1.92

## Data Availability

The data presented in this study are available on request from the corresponding authors.
